# Methods to Characterize Ricin for the Development of Reference Materials

**DOI:** 10.6028/jres.111.023

**Published:** 2006-08-01

**Authors:** Sook-Kyung Kim, Diane K. Hancock, Lili Wang, Kenneth D. Cole, Prasad T. Reddy

**Affiliations:** National Institute of Standards and Technology, Gaithersburg, MD 20899

**Keywords:** bioterrorism agent, castor bean plant, reference material, ricin toxin

## Abstract

Ricin is an abundant protein from the castor bean plant *Ricinus communis*. Because of its high toxicity and the simplicity of producing mass quantities, ricin is considered a biological terrorism agent. We have characterized ricin extensively with a view to develop Reference Materials that could be used to test and calibrate detection devices. The characterization of ricin includes: 1) purity test of a commercial batch of ricin using electrophoresis in polyacrylamide gels, 2) biological activity assay by measuring its ability to inhibit protein synthesis, 3) quantitation of protein concentration by amino acid analysis, 4) detection of ricin by an immunoassay using a flow cytometer, and 5) detection of ricin genomic DNA by polymerase chain reaction using nine different primer sets. By implementing these five methods of characterization, we are in a position to develop a reference material for ricin.

## 1. Introduction

Ricin is a highly toxic castor bean protein from the tropical plant *Ricinus communis* [1]. Doses of ricin for human lethality are not well established. There are three routes of exposure to ricin, viz., inhalation, ingestion, and injection, the latter is the most effective. An LD_50_ of 3 µg/kg body weight by inhalation and 30 µg/kg body weight by ingestion are reasonable estimates [2,3]. Extrapolating these estimates, one kg of ricin evenly spread could cause the loss of more than 2 million human lives. *R. communis* is abundant in the tropical climatic world and the annual production of castor beans is in excess of one million tons [4]. The castor beans are widely used in the production of castor oil. The pulp remaining after removal of the oil is a major source of ricin which constitutes ≈5 % of the castor bean protein. Ricin can be easily isolated in abundance from the pulp [5]. Because of the ease of isolation and high toxicity, ricin is listed as a biological terrorism agent.

Ricin is a heterodimeric protein with an observed molecular mass of ≈65 kDa. It is composed of two nonidentical subunits, ricin A chain and ricin B chain [6]. Ricin A chain is a 267 amino acids (aa) polypeptide with an apparent molecular mass of 33 kDa while ricin B chain is a 262 aa polypeptide with an apparent molecular mass of 32 kDa. Both subunits are glycosylated and the extent of glycosylation may vary based on the *R. communis* species [7]. Both the subunits A and B of ricin are synthesized by an intron-less single gene as a pre-proricin molecule with a 35 aa amino-terminal signal sequence [8]. The A and B chains are linked by a 12 aa joining peptide called the J-peptide. Two post-translational events occur in the processing of pre-pro-ricin to mature ricin. First, cleavage of the signal sequence from pre-proricin yields proricin. Next, the removal of the J-peptide from proricin yields mature ricin. The A and B chains in the mature ricin are linked by a reducible disulfide bond between cystein_259_ of the A chain and cystein_4_ of the B chain [9].

The A chain and the B chain of ricin have specific roles in culminating its toxic effects. The B chain of ricin contains two high affinity binding sites for galactose residues through which the toxin binds to the glycoproteins and glycolipids on the eukaryotic cell surface. Facilitated by the receptor mediated endocytosis, ricin enters the eukaryotic cells and upon the entry, the B chain dissociates from the A chain. Hence the role of B chain is to gain entry into the eukaryotic cells while the A chain, by the virtue of its RNA N-glycosidase activity, exerts the real toxic action. The A chain catalytically inactivates the ribosomes which are the protein synthesis machinery within the cell. The RNA N-glycosidase activity of the ricin A chain catalytically depurinates adenosine at position 4324 of the eukaryotic 28S ribosomal RNA with high specificity. Just a single ricin A molecule that enters the cytosol of a cell can inactivate over 1,700 ribosomes per minute killing the cell [10]. The catalytic nature of the ribosomal inactivation is primarily responsible for the high toxicity of ricin. Since the A chain alone cannot enter the eukaryotic cell, it is ineffective by itself.

Here we present various methods for the biochemical characterization of ricin to assess the feasibility of developing a “Reference Material” for the detection, identification, and quantification of ricin. We have characterized the purity and the subunit composition of ricin using sodium dodecylsulfate (SDS)-polyacrylamide gel electrophoresis (PAGE) [11]. We accurately determined the protein concentration by amino acid analysis and compared these results with those obtained by the commonly used method to determine the protein concentration, the Lowry method [12] using bovine serum albumin and ovalbumin as standards. We implemented a sensitive enzymatic assay for the inhibition of protein synthesis based on the inhibition of [^35^S] labeled methionine incorporation into luciferase protein [13]. We carried out an immuno-assay for the ricin toxin using a flow-cytometer based system. Finally, we isolated the genomic DNA from two varieties of castor beans and developed PCR based assay for the detection of the ricin coding sequence.

## 2. Experimental Procedures

### 2.1 Materials

Ricin, ricin A chain, ricin B chain, RCA_120_ (*Ricinus communis* Agglutinin I #L-1080) and the ricin antibody (anti-*Ricinus communis* Agglutinin I & II, goat polyclonal #AS-2084) were purchased from Vector laboratories, Burlingame, CA. Rabbit reticulocyte lysate system with luciferase mRNA was obtained from Promega corporation, Madison, WI. L-Methionine [^35^S] (1175 Ci/mmole) was the product of Perkin Elmer Life and Analytical Sciences, Shelton, CT. Plant DNA isolation kit (NucleoSpin Plant Kit) was purchased from BD Biosciences Clontech, Palo Alto, CA. Two varieties of castor beans, one variety from red leafed *Ricinus communis* plant and the other from green leafed plant, were purchased from a local nursery. Endoglycosidase H (Endo H) and N-Glycosidase F (PNGase F) were obtained from New England Biolabs, Beverly, MA. Pfu Turbo DNA polymerase was purchased from Stratagene, La Jolla, CA. Nucleotide triphosphates, acrylamide, bisacrylamide, sodium dodecylsulfate, DNase free RNase were all analytical or molecular biology grade products. Oligonucleotides, salt-free purified, were obtained from Operon, Huntsville, AL. Glass fiber filters GF/A were obtained from Whatman International Ltd. Liquefied phenol, (U.S.P. Grade) was from J.T. Baker (Phillipsburg NJ). Folin-phenol reagent, L-norvaline, and ovalbumin were from Sigma Chemical Co., St. Louis, MO. Bovine serum albumin was from Amersham, Piscataway, NJ. Amino acid hydrolyzate standard was from Hitachi (San Jose, CA). xMAP carboxylated microspheres, LabMAP Sheath Fluid, and calibration beads were purchased from Luminex Corp. (Austin, TX). Microsphere coupling reagents, EDC (1-ethyl-3-(3-dimethylaminopropyl)carbodiimide hydrochloride) and Sulfo-NHS (N-hydroxysulfosuccinimide sodium salt) were obtained from Pierce (Rockford, IL). Coupling reactions were carried out in 1.5 mL co-polymer micro-centrifuge tubes (USA Scientific, Ocala, CA). The ricin reporter antibody was biotin-labeled using the FluoReporter Mini-Biotin-XX protein labeling kit (F-6347) (Molecular Probes, Eugene, OR), and detected with streptavidin conjugated R-phycoerythrin, also from Molecular Probes. Molecular biology grade buffer components used in the Luminex coupling procedures were purchased from Sigma (St. Louis, MO). The Costar Thermowell 96-well polycarbonate plates (Corning #6509) used for the Luminex analysis were purchased from Fisher (Pittsburgh, PA).

### 2.2 Preparation of SDS-Polyacrylamide Gels

12 % polyacrylamide gels (1 mm thickness) were prepared from a stock solution containing 29.2 % acrylamide and 0.8 % bisacrylamide. The 12 % gels were prepared with 0.1 % sodium dodecylsulfate (SDS), 0.375 moles/liter Tris-HCl buffer, pH: 8.8. The gels were stacked with 4 % polyacrylamide containing 0.1 % SDS, 0.125 moles/L Tris-HCl buffer, pH: 6.8 [14].

### 2.3 Deglycosylation of the Ricin A Chain with Endo H and PNGase F

Ricin A (10 µg in 10 µL) was denatured in 1.1 µL of 10 × glycoprotein denaturation buffer (5 % SDS, 10 % β-mercaptoethanol) at 100 °C for 10 min. For digestion with Endo H, 1.5 µL of 10 × reaction buffer (0.5 moles/L Sodium citrate, pH: 5.5) was added to the denatured ricin A. One µL of Endo H containing either 10 U or 50 U was added, volume was made up to 15 µL with water, and incubated at 37 °C for 1 h. Denatured ricin A without Endo H serves as control. At the end of incubation, 5 µL of 4x SDS sample buffer (500 mMoles/L Tris-HCl buffer, pH:6.8, 715 mMoles/L 2-mercaptoethanol, 2 % SDS, 10 % glycerol, 0.05 % bromophenolblue) was added, boiled for 5 min and the reaction products were analyzed by electrophoresis on 12 % polyacrylamide gels containing SDS. For digestion with PNGase F, after denaturation of ricin A as above, 1.5 µL of 10 × reaction buffer (0.5 moles/L Sodium phosphate pH: 7.5 and 10 % NP-40 detergent) was added. One µL of PNGase F containing either 10 U or 50 U was added, volume was made up to 15 µL with water, and incubated at 37 °C for 1 hr. Denatured ricin A without PNGase F serves as control. At the end of incubation, the reaction products were separated on 12 % SDS-PAGE as described above. Proteins were stained with Coomassie blue.

### 2.4 Quantification of Ricin by Lowry Method

Lowry method [12] of protein quantification is based on the reaction of a protein with copper under alkaline conditions and subsequent reduction of the phosphomolybdic–phosphotungstic (Folin-phenol) reagent by the copper treated protein. Standard curves were generated using bovine serum albumin and ovalbumin over a range of 10 µg to 100 µg protein. Protein was incubated with 5 mL of the alkaline copper reagent at room temperature for 10 min. Folin-phenol reagent (0.5 mL) was added, immediately vortexed, and incubated at room temperature for 30 min. The developed color was read at 750 nm in a Beckman DU-650 spectrophotometer.

### 2.5 Quantification of Ricin by Amino Acid Analysis

A hydrolysis solution was prepared by adding 0.08 mL of liquefied phenol to 10 mL of concentrated HCl (12.1 N), and 60 uL was added to an equal volume of a solution containing ricin (100 µg) and norleucine (20 nanomoles). The samples were prepared in triplicate (6 × 50 mm tubes) and placed in a reaction vial containing 0.3 mL of water and 0.3 mL of the above hydrolysis solution. The vial’s valve cap was closed and the vial was evacuated (less than 1 torr), then flushed with nitrogen for three cycles of evacuations and flushing. The vial was finally evacuated, sealed and placed in an oven at 110 ºC for 22 h. After cooling the samples were reduced to dryness using a vacuum centrifuge at 45 ºC for 90 min. The dried samples were suspended in 0.2 mL of 20 mMoles/L HCl and analyzed on a Hitachi L-8800 Amino acid analyzer using post-column ninhydrin derivatization.

### 2.6 Characterization of Ricin by Enzymatic Assay

An *in vitro* translation was set up in 16.7 µL reaction volume consisting of: 11.7 µL rabbit reticulocyte lysate, 0.33 µL amino acid mixture minus methionine, 1.0 µL 35S-Methionine (1175 Ci/mmole), 0.33 µL RNasin, 0.67 µL luciferase mRNA, 1.0 µL nuclease-free water, and 1.67 µL of either ricin A, or ricin B, or ricin. One control without luciferase mRNA and a second control without any of the ricin molecules was set up in parallel. In these reactions, constant volume was maintained with the addition of water. The translation reactions were incubated at 30 °C for 90 min. A 2 µL reaction mixture was added to 98 µL of 1 mole/L sodium hydroxide containing 2 % hydrogen peroxide, mixed thoroughly and incubated at 37 °C for 10 min. At the end of the incubation, 900 µL of ice-cold 25 % trichloroacetic acid (TCA) containing 2 % casamino acids was added and incubated in ice for 30 min to precipitate the translation product ^35^S-luciferase. The precipitate was collected by vacuum filtering 250 µL of the TCA reaction mixture over a Whatman GF/A glass fiber filter. The filter was rinsed three times with 3 mL of ice-cold 5 % TCA and once with 3 mL of acetone. The filter was dried at room temperature, transferred to a scintillation vial, 5 mL of scintillation fluid was added, and the incorporation of the radioactive methionine was measured in a Packard 2200 CA liquid scintillation counter. To determine the total radioactivity present in the TCA reaction mixture, 5 µL was spotted onto the filter, dried, and counted as above. To determine the background radioactivity, 2 µL of the original 16.7 µL reaction mixture without the luciferase mRNA was processed as described above.

### 2.7 Immunoassay of Ricin-Bead Coupling, Antibody Labeling and Analysis

The Luminex 100 is a liquid microsphere-array system based on flow cytometry principles. It can be applied to protein detection using an ELISA sandwich style assay. Here a uniquely identified microsphere set is coupled to a “capture” antibody, the microsphere-capture antibody is incubated with the protein and next incubated with fluorescently labeled “reporter” antibody. Microspheres are then individually interrogated in a rapidly flowing fluid stream. As the microspheres pass between two lasers, the red laser identifies the specific microsphere of the array and the green laser identifies the fluorescently labeled “reporter” antibody bound to the protein (analyte).

Working in subdued light, the microspheres for this study were prepared by coupling the carboxylated microspheres with the Vector polyclonal ricin antibody using carbodiimide chemistry according to the protocols suggested by Luminex. The coupling reaction was carried out at antibody concentrations of 24, 100, 170, and 250 µg/mL in 0.05 moles/L MES (2-[N-morpholino]ethane sulfonic acid), pH 6.0. The coupled microspheres were suspended in a blocking storage buffer (0.01 mole/L phosphate, 0.138 mole/L NaCl, 0.0027 mole/L KCl, pH 7.4, with 10 mg/mL bovine serum albumin and 0.05 % (w/v) NaN_3_) and bead counts were performed on a diluted sample using a Hausser Scientific Bright-Line Hemocytometer (# 15170-172, VWR, Planfield, NJ). The “reporter” antibody was prepared by biotinylating an additional portion of the same polyclonal Vector antibody, following the protocols provided with the Molecular Probes Biotin-XX Protein Labeling Kit, yielding a 1.4 mg/mL biotinylated-antibody stock solution.

The ricin analyses were performed in subdued light by adding a suspension of 1,000–50,000 microspheres in 14 µL of blocking storage buffer to either microfuge tubes or to wells of a 96-well plate. Twenty µL of a target analyte solution (e.g. ricin) also in blocking storage buffer was added to the microspheres and the samples were mixed by gently vortexing for 20 minutes on a platform head (Vortex-Genie2, Daigger, Vernon Hills, IL). Thirty µL of diluted biotinylated-antibody stock solution (13 µg/mL) was added to each sample and samples were incubated in the dark for 20 minutes. Ten µL of a streptavidin-phycoerythrin dye complex was added to each sample, and incubated for 10 minutes, thus labeling the biotinylated antibody with a sensitive fluorescent moiety. Then the 96-well plate was placed in the Luminex instrument and the samples were analyzed, interrogating between 100 and 250 microspheres for each target analyte.

### 2.8 Isolation of Genomic DNA From *Ricinus communis* Seeds

DNA was isolated using the kit and the protocol provided by Clontech. One bean from a red-leafed plant (470 mg) and one bean from green-leafed plant (655 mg) were crushed in a mortar and further homogenized with 600 µL of Buffer C1 (composition for all of the buffers described in this section is not available from the manufacturer of the NucleoSpin nucleic acid purification kit, Clontech). The suspension was transferred to a 1.5 mL microcentrifuge tube and treated with 40 µL of 1 mg/mL RNase for 10 min at room temperature. The suspension was further incubated at 65 °C for 30 min and centrifuged at 16,000 × g for 15 min. The clear supernatant was transferred to a 15 mL polystyrene tube and 600 µL of Buffer C4 and 400 µL of 95 % ethanol were added. The mixture was thoroughly vortexed for 30 s. The sample was applied to a NucleoSpin column and spun at 16,000 × g for 1 min. The column was successively washed with 500 µL of Buffer CW, and 500 µL of Buffer C5, and again with 500 µL of Buffer C5 to remove contaminants. The column was centrifuged for an additional 1 min. The DNA was eluted successively with two 100 µL volumes of pre-warmed Buffer CE. Both of the eluates were analyzed by electrophoresis on a 1 % agarose gel in TAE buffer (40 mmoles/L Tris-acetate, pH: 7.5, 1 mmole/L EDTA) (14).

### 2.9 Polymerase Chain Reaction

Several segments of the ricin coding sequence were amplified by PCR using the primer sets shown in Table 3. The PCR mixture contained in a 25 µL final volume: 2.5 µL of 10 × PCR buffer, 2.5 µL 2 mmole/L dNTP’s, 200 ng of genomic castor bean DNA, 100 ng each of forward and reverse primers, 0.5 µL Pfu Turbo DNA polymerase (2.5 U). Water was added to make the volume up to 25 µL. Amplification conditions were: 95 °C for 60 s for initial melting of DNA, followed by 30 cycles of amplification each cycle consisting of melting at 95 °C for 60 s, annealing at 50 °C for 60 s, and polymerization at 72 °C for 60 s. Polymerization was continued at the end for 10 min at 72 °C. An independent amplification was performed under identical conditions except for the changes in the annealing temperature from 50 °C to 60 °C. Thermocycling was conducted in the Applied Biosystems GeneAmp PCR System 9700.

## 3. Results and Discussion

### 3.1 Characterization of Ricin A Chain, Ricin B Chain, and Ricin by SDS-PAGE

**Fig. 1:**
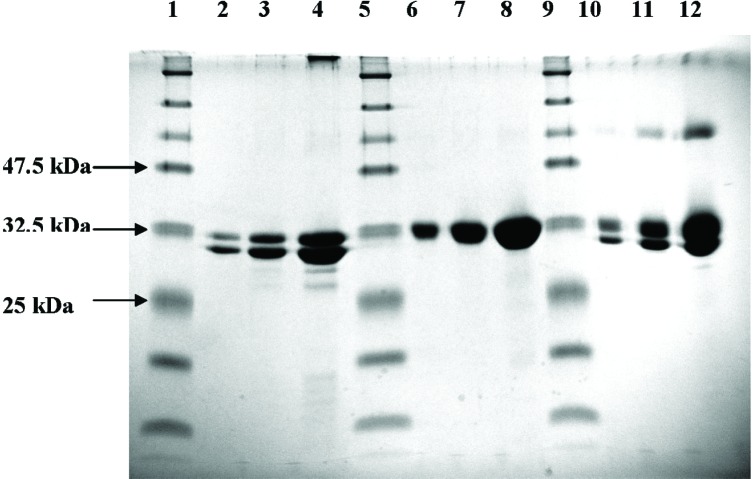
SDS-PAGE analysis of purified ricin A chain, ricin B chain, and ricin. Purified preparations of ricin A chain, ricin B chain, and ricin whole molecules were analyzed on a 12 % acrylamide gel. Proteins were stained with Coomassie blue. Lanes 1, 5, and 9: molecular mass markers. Lanes 2, 3, and 4: 1 µg, 3 µg, and 10 µg of ricin A chain protein, respectively. Lanes 6, 7, and 8: 1 µg, 3 µg, and 10 µg of ricin B chain protein, respectively. Lanes 10, 11, and 12: 1 µg, 3 µg, and 10 µg of ricin protein, respectively.

Our initial characterization of ricin was by SDS-PAGE to check for purity and migration behavior on the acrylamide gels. Various concentrations of ricin A chain (Fig. 1, lanes 2–4), ricin B chain (lanes 6–8), and ricin AB whole molecule (lanes 10–12) were run on 12 % SDS-Polyacrylamide gel. It is interesting to note that ricin A chain alone exhibits two Coomassie stainable protein bands. The upper protein band termed ricin A1, and the lower protein band termed ricin A2 are of ≈40 % and ≈60 % intensity, respectively. The appearance of two protein bands for ricin A chain is a consequence of differential glycosylation, not due to a contaminating protein. The existence of two discrete protein bands in a purified ricin A preparation has been observed by Fulton et al [6] and our data confirm the reported results. Ricin B chain migrates as a single protein band which suggests no detectable heterogeneity in the glycosylation of this subunit. The ricin whole molecule was separated into two protein bands. The upper band in ricin is a mixture of ricin A1 and ricin B chains. The lower protein band corresponds exclusively to ricin A2.

### 3.2 Deglycosylation of Ricin A Chain

**Fig. 2:**
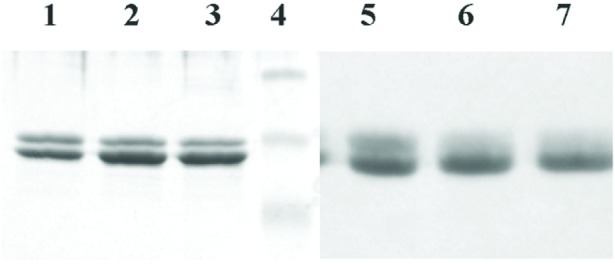
Deglycosylation of the ricin A chain with Endo H and PNGase F. 12 % SDS-PAGE analysis of Ricin A chain products following deglycosylation with either Endo H or PNGase F enzymes. Lane 1: control (no Endo H), lanes 2 and 3: 10 units and 50 units of Endo H, respectively, lane 4: Molecular mass markers (upper band, 47.5 kDa, middle band, 32.5 kDa, and lower band, 25 kDa), lane 5: control, no PNGase F, lanes 6 and 7: 10 units and 50 units of PNGase F, respectively.

As pointed out above, ricin A chain exhibits heterogeneity due to the extent and the type of post-translationally added sugar residues, a phenomenon called glycosylation. We therefore performed deglycosylation of ricin A chain with Endoglycosidase H (Endo H) and N-Glycosidase F (PNGase F). Endo H cleaves within the chitobiose core of high mannose and some hybrid oligosaccharides from asparagine linked proteins. PNGase F is an amidase which cleaves between the innermost GlcNAc and asparagine residue of the glycoproteins. As shown in Fig. 2, deglycosylation with Endo H resulted in some degree of deglycosylation as the intensity of the ricin A1 band decreased and there was a corresponding increase in the intensity of the ricin A2 band (compare lane 1 without enzyme to lane 2 with 10 U of enzyme and lane 3 with 50 U of enzyme). However, Endo H did not completely remove the carbohydrate moieties from the ricin A chain. On the other hand, deglycosylation by similar treatment with PNGase F resulted in nearly complete removal of sugar residues from the ricin A1 chain (compare lane 5 without enzyme to lane 6 with 10 U of enzyme and lane 7 with 50 U of enzyme, Fig. 2).

### 3.3 Quantification of Ricin by Lowry Method

The Lowry procedure is one of the widely used protein quantification assays, being first described in 1951 [12]. Under alkaline conditions, copper complexes with a protein and turns from the reduced cuprous state to the oxidized cupric state. When Folin-phenol (phosphomolybdic-phosphotungstic) reagent is added, the Folin-phenol reagent binds to the copper-protein oxidized complex and the reagent is slowly reduced and changes color from yellow to blue. Although the Lowry method is a classical assay, it is known to be less reliable than some other protein assays as it is more subject to interference by a wide variety of chemicals commonly used in the purification of proteins. Among the chemicals reported to interfere with the Lowry procedure are dithiothreitol, mercaptoethanol, EDTA, HEPES, Tricine, Tris, and Triton X-100. There is also much protein-to-protein variation in the intensity of color development. Hence the protein concentration determined by this assay may vary depending on the protein used in the assay’s standard curve.

We determined the protein concentration in ricin A chain, ricin B chain, and ricin whole molecule by the standard Lowry method [12] using both bovine serum albumin and ovalbumin as standard proteins. The results are presented in Table 1. The concentration of ricin B chain matched closely with the concentrations provided by the Vector laboratories when calculated using BSA or ovalbumin standard curve. The concentration of ricin A chain was 50 % higher using BSA and 35 % higher with ovalbumin as the standard. The concentration of ricin protein was 23 % higher using BSA and 8 % higher with ovalbumin as the standard. In all the three cases, protein concentrations calculated with the ovalbumin standard are closer to the concentrations provided by the supplier Vector laboratories. We therefore quantified the protein content of ricin A and ricin samples by amino acid analysis for greater accuracy and precision.

### 3.4 Quantification of Ricin by Amino Acid Analysis

The results of measuring the concentration of the ricin sample based on recovery of individual amino acids is shown in Table 2. The number of amino acid residues in the A-chain, B-chain, and ricin are based on the amino acid sequence obtained from Swiss-Prot (accession number P02879). The amino acid numbers and the molecular masses were calculated using the secreted form of ricin with the prepropeptide and linking peptide removed from the ricin A and B chains [15]. Only those amino acids that are considered to be well recovered after protein hydrolysis were used to calculate the concentration data shown in Table 2 [16,17]. The results are consistent with the expected amino acid composition of ricin. A standard preparation of bovine serum albumin was also hydrolyzed and the expected amino acid composition was also obtained (results not shown). The amino acid measurements were done in triplicate on the same sample of ricin. The analysis was repeated approximately 9 months later on the same sample (stored at 4 °C) and the results agree with the error of the measurements.

The calculated molecular masses for the A-chain, B-chain, and ricin are 29,908.8, 28,967.6, and 58,874.4 (subtracted 2 for a disulfide bond formation) based on the amino acid sequences. These molecular masses do not include the mass added to the protein due to glycosylation. The average value for the ricin sample is 67.2 nmol/mL based on the data shown in Table 2. This molar concentration corresponds to 3.96 mg of ricin protein/mL for the ricin sample. This value does not include the additional mass due to the glycosylation of ricin.

### 3.5 Effect of Ricin A, Ricin B, and Ricin on Protein Synthesis

The ricin A chain and the ricin B chain have specific roles in the manifestation of ricin’s toxic effect on eukaryotic cells. The primary role of the B chain is to gain entry into the eukaryotic cells. The high affinity of the B chain’s galactose residues to the glycoproteins and glycolipids on the eukaryotic cell surface facilitates the translocation of the ricin molecule into the cytosol. Upon the entry, the B chain dissociates from the A chain. The A chain, by the virtue of its highly specific RNA N-glycosidase activity, depurinates adenosine at position number 4324 of the eukaryotic 28S ribosomal RNA, thus inhibiting protein synthesis. A single ricin A molecule that enters the cytosol of an eukaryotic cell can inactivate over 1,700 ribosomes per minute killing the cell [10]. On the basis of this activity we designed an *in vitro* cell-free protein synthesis to measure the effect of ricin A chain, and the whole ricin AB molecule. The ricin B chain was included as a control since it is not expected to inhibit protein synthesis by itself.

**Fig. 3:**
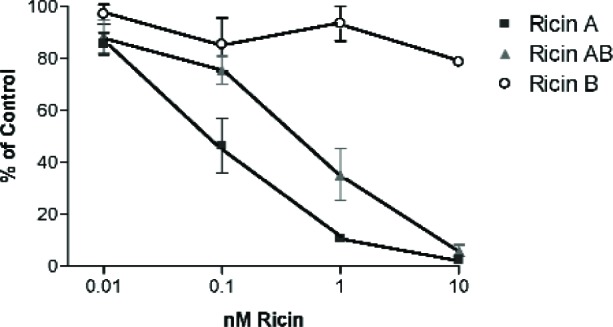
Effect of ricin A, ricin B, and ricin on protein synthesis. The effect of various concentrations of ricin A, ricin B, and ricin on the *in vitro* translation of Luciferase mRNA by the rabbit reticulocyte lysate was carried out as described in the “Experimental Procedures”. The incorporation of [^35^S]-methionine was measured and plotted as the % remaining activity on the Y-axis. A 100 % activity represents the total incorporation of [^35^S]-methionine in the control translation assay without any of the ricin molecules.

We monitored the protein synthesis by measuring the incorporation of radiolabeled [^35^S]-methionine into the luciferase protein by an *in vitro* translation of luciferase mRNA as a function of the ricin A chain, ricin B chain and the ricin AB whole molecule concentrations. The results of these measurements are presented in Fig. 3. Ricin A chain was found to be more potent than the whole ricin molecule in inhibiting luciferase synthesis. At 0.1 nmole/L and 1.0 nmole/L concentrations, the A chain caused 60 % and 85 % inhibition of protein synthesis, respectively, compared with 25 % and 75 % inhibition by the whole ricin AB molecule at the same concentrations. At 10 nmole/L concentration both ricin A and ricin AB inhibited the protein synthesis by greater than 90 %. Under our *in vitro* assay conditions, it appears that the ricin B chain does not completely dissociate from the A chain. Since the A chain must be reductively cleaved from the B chain to expose the active site [18,19] the whole ricin molecule is less toxic than the free ricin A chain. Ricin B shows only a slight inhibitory activity which may be due to some contamination with ricin A during its purification.

### 3.6 Detection of Ricin with Antibody

**Fig. 4:**
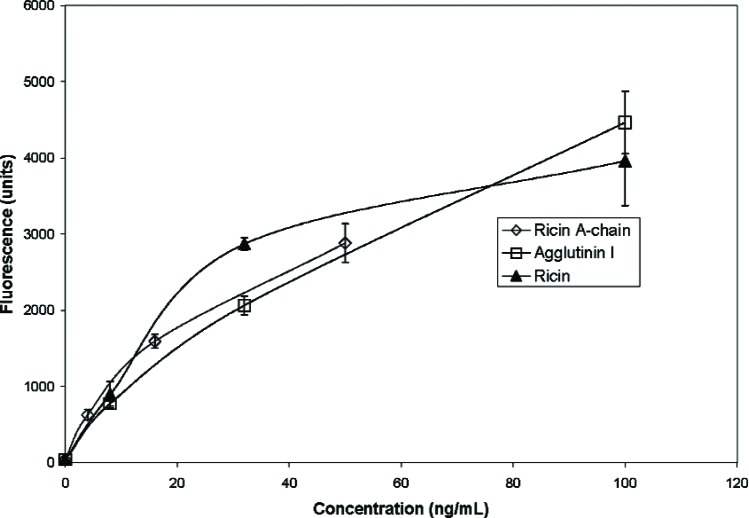
Sensitivity of the immunoassay for ricin, agglutin I, and ricin A-chain. Average fluorescence readings from antibody-coated microspheres, hybridized with varying concentrations of ricin, agglutinin I, or ricin A chain, and detected with biotinylated antibody complexed with streptavidin-R-phycoerythrin.

**Fig. 5:**
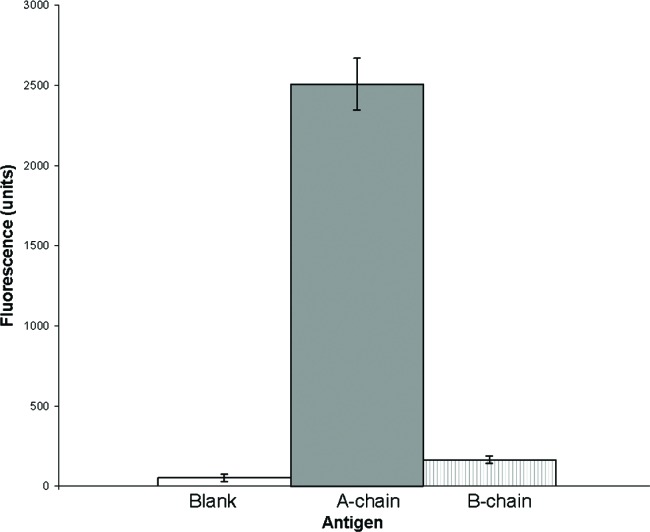
Specificity of the Luminex immunoassay for Ricin A and B chains. Average fluorescence of Agglutinin I and II antibody-coated microspheres incubated with either Ricin A chain, ricin B chain, or a control sample (water), and then with biotinylated-antibody and finally with streptavidin-R-phycoerythrin. Analyte concentrations were 50 ng/mL.

An ELISA-style “sandwich” immunoassay developed for Luminex system analysis was used for detection and quantification of ricin. Incubations of the “capture” antibody-conjugated-microspheres with each analyte (ricin A chain, ricin B chain, agglutinin I, and the whole ricin molecule), followed by biotin-labeled-“reporter”-antibody and streptavidin-phycoerythrin produced the “sandwich.” As individual microspheres were interrogated between two lasers in a rapidly flowing stream, the red laser identified the specific member of the microsphere-array and the green laser identifies the amount of green fluorescent phycoerythrin bound to the microsphere via the biotin-labeled-antibody. In the absence of antibody specific to the analyte, the analyte is not captured, and the phycoerythrin is not bound to the microsphere “sandwich”, hence no positive reaction is reported. Using the goat polyclonal antibody developed to the ricin agglutinin I and II as both the “capture” and “reporter” antibody gave strong positive reactions to the whole ricin, agglutinin I, and ricin A chains as shown in Fig. 4. Each of these toxic species were detected at 3 ng/mL. In comparison to the ricin A chain the antibody binds only marginally to the nontoxic ricin B chain (Fig. 5). This marginal binding may be nonspecific in nature or it may reflect contamination of the ricin B chain with ricin A chain as was also suggested by the slight inhibitory activity observed in the protein synthesis assay. This Luminex immunoassay using the tested polyclonal antibody provides a sensitive, specific assay for these toxic ricin moieties.

### 3.7 Detection of Ricin Coding Sequence by Polymerase Chain Reaction (PCR)

**Fig. 6:**
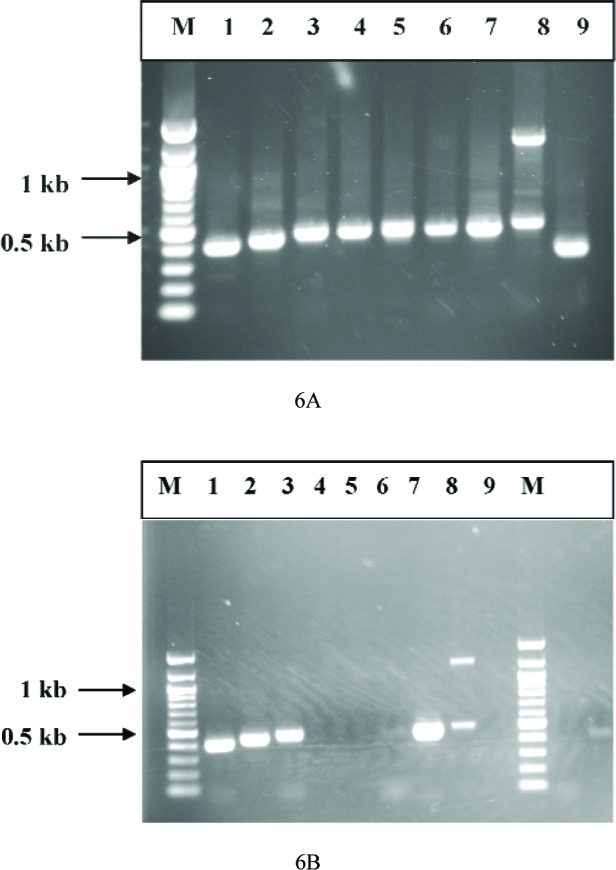
Detection of ricin genomic DNA by polymerase chain reaction (PCR). The purification of genomic DNA from a single castor bean was described in the “Experimental Procedures”. Genomic DNA from a single castor bean was amplified using nine different primer sets (Table 3) and 2 annealing temperatures. Agarose gels (1 %) of the amplified products are shown. 4A: annealing temperature 50 °C, 4B: annealing temperature 60 °C.

The cloning and sequencing of the ricin gene has shown that it is an intron-less sequence [8], a rare and an unusual feature of the eukaryotic gene. The advantage of amplifying an intron-less sequence is that the amplicon size can be predicted from the positions of the forward and reverse oligonucleotide primers. Hence, we used a total of nine primer sets for the amplification of various segments of the coding sequence of ricin (Table 3). The PCR products were analyzed on a 1 % agarose gel and the number of base pairs in each amplicon was estimated based on the molecular markers that were run in parallel. PCR amplification was conducted at two annealing temperatures, 50 °C, and 60 °C using genomic DNA from a single castor bean seed from each of two species of *Ricinus communis*. The results of the amplification of DNA isolated from one castor bean are presented (Fig. 6A–B). PCR performed at the annealing temperatures 50 °C (Fig. 6A) produced amplicons of expected sizes of ricin genomic DNA with all the 9 primer sets. The results for the other species of castor bean were the same and these data are not shown. Amplifications performed at the annealing temperature of 60 °C produced amplicons only with primer sets 1, 2, 3, 7, and 8 but no amplicons were seen with primer sets 4, 5, 6, and 9. An interesting feature of amplification with the primer set 8 was that an unexpected size amplicon of about 1300 base pairs was produced at both 50 °C and at 60 °C annealing temperatures. Amplification at higher annealing temperature results in specific binding of an oligonucleotide to the genomic DNA and concomitant amplification of correct size amplicons.

## 4. Conclusions

In order to detect and quantify ricin, a highly toxic and potential biological terrorism agent, we have explored the feasibility of developing a “Reference Material” for ricin by measuring several of its biochemical properties. We measured the biological activity of ricin and showed that ricin inhibits protein synthesis. We performed immunoassays to detect ricin at subfem-tomole levels. We developed a polymerase chain reaction assay to detect ricin’s genomic DNA. By implementing and developing these assays, we are in a position to carryout analyses required for the certification of a “Reference Material” for ricin.

## Figures and Tables

**Table 1:** Comparison of protein concentration determined with bovine serum albumin (BSA) and ovalbumin as standards

Ricin preparation	Vector labs (mg/mL)	Lowry Method (mg/mL)	
		BSA	Ovalbumin
Ricin	5.0	6.14 ± 0.22	5.41 ± 0.37
Ricin A	1.0	1.53 ± 0.17	1.35 ± 0.20
Ricin B	1.0	1.14 ± 0.005	1.04 ± 0.005

**Table 2:** Concentration of ricin determined by well-recovered amino acids

Amino Acid	A-chain Residues/mole	B-chain Residues/mole	Ricin Residues/mole	Ricin Concentration First determination (nmol/mL)*	Ricin Concentration Second determination (approx. 9 months later) (nmol/mL)*
Aspartic acid	8	17	25	65.3 ± 0.7	69.1 ± 0.8
Asparagine	17	21	38		
Glutamic acid	15	5	20	68.6 ± 1.5	70.8 ± 0.6
Glutamine	14	15	29		
Glycine	17	20	37	65.5 ± 1.0	68.4 ± 1.1
Alanine	24	15	39	65.2 ± 0.8	66.9 ± 2.4
Leucine	22	24	46	68.1 ± 0.1	72.1 ± 0.4
Lysine	2	7	9	65.0 ± 0.9	65.7 ± 0.7
Arginine	21	13	34	64.1 ± 0.4	65.2 ± 0.5

* During hydrolysis asparagine and glutamine are converted to the corresponding acids. Amino acid residues for A-chain, B-chain, and ricin were obtained Swiss-Prot (http://ca.expasy.org/sprot/) using Accession number P02879.

**Table 3:** Sequence of oligonucleotides for the amplification of Ricin

(Gene Accession Number X52908)
**Primer set 1**
Forward Primer @ nt 311: 5′ATGAAACCGGGAGGAAATAC 3′
Reverse Primer @ nt 720: 5′ TCTGCATCTTCCTGATTGTCAGG 3′
**Primer set 2**
Forward Primer @ nt 455: 5′ GCGGGTGCCACTGTGCAAAGCTACAC 3′
Reverse Primer @ nt 900: 5′ GGAAGCTGAGTGCCACCAGTAC 3′
**Primer set 3**
Forward Primer @ nt 601: 5′ CTCAAATCATGCAGAGCTTTCTG 3′
Reverse Primer @ nt 1080: 5′ TTAGACTCTTGAATTGCAGTGG 3′
**Primer set 4**
Forward Primer @ nt 781: 5′ TTATGATAGACTTGAACAAC 3′
Reverse Primer @ nt 1260: 5′ ACATCAGCATTAAAATTTGG 3′
**Primer set 5**
Forward Primer @ nt 961: 5′ ATATATTGAGGGAGAAATGCGC 3′
Reverse Primer @ nt 1440: 5′ CACTTTCCATTAGATCGAATAG 3′
**Primer set 6**
Forward Primer @ nt 1141: 5′ GTACGATGTGAGTATATTAATCCC 3′
Reverse Primer @ nt 1620: 5′ TTGGTTTGCACTGTAAGTGTGG 3′
**Primer set 7**
Forward Primer @ nt 1321: 5′ TAGGGATGGAAGATTCCACAACGG 3′
Reverse Primer @ nt 1800: 5′ GGACGTATTGAACCATCTGC 3′
**Primer set 8**
Forward Primer @ nt 1501: 5′ TGCAACTGATGCCACCCGCTGGC 3′
Reverse Primer @ nt 2016: 5′ TTTGGGTCACCATGGAGAGGGTAAAG 3′
**Primer set 9**
Forward Primer @ nt 1681: 5′ CATTGTTGGGCTATATGGTCTG 3′
Reverse Primer @ nt 2041: 5′ TCAAAATAATGGTAACCATATTTGG 3′
